# Awareness of age-related change, chronological age, subjective age and proactivity: An empirical study in China

**DOI:** 10.3389/fpsyt.2022.915673

**Published:** 2022-09-29

**Authors:** Wanli Zhang, Stephen Wood

**Affiliations:** ^1^Beijing Institute of Technology, Beijing, China; ^2^Yangtze Delta Region Academy of Beijing Institute of Technology, Jiaxing, China; ^3^University of Leicester School of Business, Leicester, United Kingdom

**Keywords:** awareness of age-related change, chronological age, subjective age, task proactivity, development proactivity, organization proactivity

## Abstract

An aging workforce and the increasing value placed on employees' proactivity are two important features of current workplaces. We address the extent to which this proactivity is affected by age and aging. The study has two objectives. First, it aims to validate the concept of awareness of age-related change (AARC) in the Chinese context. Second, it compares the explanatory power of AARC with that of chronological age and subjective age in predicting three types of proactivity: task proactivity, development proactivity and organization proactivity. We used the ten-item AARC instrument in a survey of teachers (*n* = 421, mean age = 41.0) in China, and validated its content by comparing the responses of a subsample of these teachers (*n* = 33, mean age = 42.5) to questions asked in a semi-structured interview. This confirmed the validity of the instrument's content, and its applicability beyond North America and Europe, in a Chinese context. We then show that awareness of positive and negative age-related changes (AARC-Gains and AARC-Losses) are, respectively, positively and negatively associated with the three types of proactivity, and that they are better predictors than chronological age and subjective age. The research adds weight to challenges to negative age stereotyping—-that older employees are set in their ways and less proactive—-and to claims about the value of AARC for measuring aging, by showing that this factor can predict outcomes beyond health and the concerns of older adults.

## Introduction

Awareness of age-related change (AARC) is a concept introduced by Diehl and Wahl (1) to describe “all those experiences that make a person aware that his or her behavior, level of performance, or ways of experiencing his or her life have changed as a consequence of having grown older” (p. 340). It entails two dimensions, reflecting positive and negative changes, respectively. Positive changes indicate perceived gains including health and physical functioning, cognitive functioning, interpersonal relations, social cognitive and social emotional functioning, and lifestyle and engagement, while negative changes show the perception of losses in such areas. “With my increasing age, I realize that I pay more attention to my health” is an example of a statement related to AARC-Gains, while “With my increasing age, I realize that I have less energy” relates to AARC-Losses.

This two-dimensional structure has been confirmed by previous studies (2, 3). Significantly, this has been true regardless of the number of items used in the instrument—-there are 189-item (2), 50-item (4), 20-item (5) and 10-item (6) versions. Empirical research has shown that AARC-Gains leads to desirable outcomes, while AARC-Losses is related to undesirable outcomes. The majority of studies of its concomitants have concentrated on wellbeing (3–5, 7–12). For example, Brothers et al. (3) found AARC-Gains to be positively associated with life satisfaction, their measure of wellbeing (β = 0.0 4, *p* ≤ 0.05), whereas negative age-related change had a negative association (β = −0.08, *p* ≤ 0.001). Other outcomes considered in the research are cognitive interference and performance (13–15), goal adjustment (16), and health (17). For example, Wilton-Harding and Windsor reported that the dimensions of AARC are, respectively, positively and negatively related to goal re-engagement [the identification and pursuit of alternative goals – (18)], (AARC-Gains: β = 0.49, *p* ≤ 0.001; AARC-Losses: β = −0.26, *p* ≤ 0.001).

### AARC, subjective age and chronological age

AARC is conceptually different from other age-related concepts such as chronological age – the age of a person as measured from birth to a given date – and subjective age – how old a person feels (19–21). The correlations between AARC and chronological age are sufficiently low to suggest that they are discrete concepts [(7), *r* = 0.05–0.22; (3), *r* = 0.14–0.27]. Correlations between the two dimensions of AARC and subjective age are also low [(8), *r* = −0.04 to −0.32; (4), *r* = −0.10 to 0.28; (6), *r* = −0.24 to 0.26; (15), *r* = −0.06 to 0.22], indicating a conceptual difference between the two constructs. AARC is a measure of perceived aging and not just a different way of measuring subjective age than the conventional way of assessing how old people feel. It is argued that the reason for the distinctiveness of the concept of AARC is that it captures “the specific psychological and behavioral situations that make individuals aware of their age,” which offers a way to “unpack” the “black box” of subjective age [(2), p. 8]. It is a multidimensional age-related construct that emphasizes that aging is not always a negative process, and that people can play a positive role in influencing their aging process (22), while subjective age is generally considered as a unidimensional concept The only three studies that compare AARC with chronological age and subjective age (3, 8, 9) concluded, on the basis of regression coefficients, that both dimensions of AARC are better predictors of satisfaction with life, goal engagement, goal disengagement, and depressive symptoms than either chronological age or subjective age.

### Proactivity

The research so far has been predominantly conducted in the USA, UK and Germany, involved samples with mean ages around the conventional retirement age of 65 (with limited coverage of employed people), and were concerned with physical or mental health outcomes (17). We report a study of teachers working in Chinese schools, with a mean age of 41, that concentrates on AARC as a potential predictor of employee proactivity, a work-related outcome which, akin to Bindl and Parker (23), we define as self-initiated and future-oriented behavior that aims to change employees themselves or their work environment. Following Strauss and Parker (24), we consider three types of proactivity: (a) task proactivity, which focuses on how employees display initiative to complete and improve performance of their core tasks; (b) development proactivity, which relates to how people display initiative to achieve self-development; and (c) organization proactivity, which is based on exercising initiative to change an organization and help other people in the workplace.

The importance of proactivity for organizational performance has been widely emphasized in the literature on management theory (25, 26), quality management (27) and work psychology (23, 28, 29). Since the start of the century, there has been a perception among management theorists and researchers that employers are increasingly requiring employees to be proactive (30–32). Innovation is not simply about big new technological developments, but also about incremental changes that can be fostered by emphasizing the need for continuous improvements. More recently, the World Bank (33) reported that demand for employees with high non-routine cognitive and socio-behavioral skills has increased since 2001 in both industrialized and developing countries, from 33% to 41% and 19% to 23%, respectively, implying an increasing demand for employee proactivity. This chimes with a 2014 report on the future of work in the UK, which highlighted that problem-solving, adaptability, self-directed learning and creative work would be at a premium in future (34). The importance of proactivity is also stressed in other countries including the USA and China. Based on a survey in the US in 2016, 49% of human resource professionals stated that displaying initiatives is one of the most important skills for entry-level job candidates (35). In 2021, Business Linkedin released a report that problem-solving, communication, teamwork, and strategic thinking are the most valuable skills emphasized by Chinese firms when recruiting (36).

### Research aims and hypotheses

This study first validates and assesses the applicability of the concept of AARC to the Chinese context by examining whether the data from the 10-item questionnaire conform to the two-factor structure, and by comparing responses to questions on aging administered in interviews with questionnaire respondents. Second, we test whether AARC has the strongest predictive power for proactivity among these three age-related constructs. We expect this to be the case, as the concept of AARC captures the heterogeneity of experiences of aging.

Prior studies have found varying relationships between proactivity and chronological age. Specifically, associations between chronological age and task proactivity have been reported as positive (37–39), insignificant (40–43) and negative (44, 45). Examination of the relationship between chronological age and development proactivity have also given inconsistent results, although no positive ones have been found, as the associations found were either insignificant (24, 38) or negative (46, 47). The relationship between organization proactivity and chronological age has been found to be either positive (48) or not significant (24, 49). Only two studies explore the relationship between subjective age and task proactivity, both finding it to be negative (50, 51).

In light of these inconsistent results, we might expect AARC will be a better predictor of proactivity than chronological age or subjective age for all proactivity types. However, it is possible that a low correlation between age and proactivity could be the result of their relationship being mediated by AARC-Gains and AARC-Losses, with the two processes canceling each other out. In past studies, chronological age has been found to be positively related to AARC-gains and AARC-Losses (3, 7, 8). We therefore assess whether the two dimensions of AARC could mediate the relationships between chronological age and proactivity types when we examine the relationships amongst the three age-related concepts and proactivity.

## Methodology

The study addresses two research questions: (1) is the 10-item scale of AARC applicable to the Chinese context? (2) what are the relationships among chronological age, subjective age, AARC and work-related proactivity?

### Method

In common with other investigations of awareness of age-related change, we used a self-administered questionnaire to collect data on this measure and our main dependent variable, proactivity. We conducted the questionnaire in eight schools located in southeast China, comprising three primary schools, two middle schools, two high schools and one college. The first author personally handed the questionnaires to teachers either in their office or at staff meetings and collected them immediately after they were completed. Of 438 questionnaires collected, 421 (96.1%) yielded useable data, while the other 17 had too many missing data items to be useable.

Teachers were considered a good choice for our study as they have sufficient autonomy to allow them to exercise initiative, autonomy having been identified as a correlate of proactivity (52). Additionally, since the arrangements for teachers in China is not dissimilar to those of other professions in terms of pensions, employment contracts, continuing professional development expectations, working practices, and the hierarchical nature of the organizations, the results are generalizable to other professions. Furthermore, working age people have been neglected in research on age-related change and the significance of focusing on middle age people has been highlighted by Diehl et al. (53), as they argue “they are at a life stage when they become aware of age-related changes” (p. 10).

To validate the data, we interviewed 33 teachers who had completed the questionnaire. Respondents were asked when they completed the questionnaire if they would be willing to be interviewed to expand on the topics in the questionnaire. Twenty-two interviewees were acquired this way, and 11 more were obtained by asking school principals to suggest further potential interviewees. The interviews were conducted in-person in meeting rooms at the schools, and were audio recorded and transcribed. They lasted between 40 and 90 min, with a mean time of 52 minutes.

### The sample

Table 1 displays the characteristics of the samples for both the questionnaire and interview elements. The 421 participants who gave valid questionnaire responses had a mean age of 41.0 (SD = 8.5), and were aged between 20 and 65. 72.7% were female and 27.3% male and 87.4% had a bachelor's degree or higher qualification. Most respondents (83.4%) were classroom teachers, the remainder being in management positions. The 33 interviewees were aged between 23 and 62 (mean age = 42.5, SD = 10.5), with 11 male and 22 female participants. Again, the majority of interviewees had a bachelor's degree or higher qualification (32 people). Eighteen interviewees were classroom teachers, seven were middle-level managers and eight were top-level managers.

**Table 1:** Demographic characteristics of participants in the two elements of the study.

**Variables**	**Questionnaire participants**	**Interview participants**
**Age (mean, SD, range)**	41.0, 8.9, 20–65	42.5, 10.5, 23–62
**Age band (%) 20–30**	15.9	12.1
**31–40**	28.1	24.2
**41–50**	46.3	33.3
**51–60**	8.3	24.3
**61–65**	1.4	6.1
**Gender (%)**
Male	27.3	33.3
Female	72.7	66.7
**Education level (%)**
Below bachelor's degree	12.6	3.0
Bachelor's degree	80.0	75.8
Master's degree and above	7.4	21.2
**Job position (%)**
Classroom teacher	83.4	54.5
Middle-level manager	10.7	21.2
Top-level manager	5.9	24.3

### Measures

*AARC* (AARC-Gains: α = 0.76; AARC-Losses: α = 0.68) was assessed using the ten-item scale developed by Kaspar et al. (6). This involves asking “With my increasing age, I realize that…”, combined with ten different items. Sample items for AARC-Gains and AARC-Losses, respectively, were “I pay more attention to my health” and “I have less energy.” The response format was: 1 = “Strongly disagree,” 2 = “Disagree,” 3 = “Neither agree nor disagree,” 4 = “Agree,” and 5 = “Strongly agree.” Factor analysis confirmed the construct's two-dimensional structure (see Section 2.4).

Chronological age was measured through the question “What is your age?”

Subjective age was measured by asking two questions. The first is “Which age group do you FEEL you really belong to?” The response scale is divided into 10-year age groups: 20–30, 31–40, 41–50, 51–60, 61–70, 71–80, and 81 and over. The second question is “Can you specify how old you actually feel?” If respondents did not give a specific subjective age in answer to the second question (32.8% of respondents did not give a figure), their subjective age was imputed as the middle number of the range indicated in the first question (e.g., if an individual felt they belonged to the 31–40 age group, their subjective age was taken as 35).

Task proactivity (α = 0.67) was measured by asking respondents three questions, all starting: “Over the past 2 years, to what extent have you…”, followed by three different statements: “Initiated better ways of doing your core tasks?” “Come up with ideas to improve the way in which your core tasks are done,” and “Made changes to the way your core tasks are done.” These were adopted from Griffin et al.'s (26) scale. A five-point Likert response scale was used for this and the two other proactivity types thus: 1 = “Not at all,” 2 = “Little,” 3 = “Somewhat,” 4 = “Much,” and 5 = “A great deal.”

Development proactivity (α = 0.82) was measured using Strauss and Parker (24) three-item scale starting with a statement, “Over the past 2 years, to what extent have you…”, the items are “Developed knowledge and skills in tasks critical to your future work life,” “Developed skills which may be needed in the future” and “Gained experience in a variety of tasks to increase your knowledge and skills.”

Organization proactivity (α = 0.85) was measured by combining items from Griffin et al. (26), Zacher et al. (54) and Wells et al. (55). Again starting with a statement, “Over the past 2 years, to what extent have you…”, the items are “Involved yourself in changes that are helping to improve the overall effectiveness of the organization,” “Come up with ways of increasing efficiency within the organization,” “Made suggestions to improve the overall effectiveness of the organization (e.g., by suggesting changes to administrative procedures),” “Been more concerned with building up the next generation of employees than you were a few years ago,” and “Been more devoted to passing along the knowledge you have gained than you were a few years ago.”

Control variables used were gender, organizational tenure and management position. Gender was measured using the question: “What is your gender?” Possible responses were “Male” and “Female.” Organizational tenure was measured by asking: “How long have you been working in this school?” Management position was assessed by asking “What is your job position?” The possible responses were: “Classroom teacher,” “Middle-level manager” and “Top-level manager.” Management position is coded as one for middle- or top-level managers, and zero for classroom teachers.

Four questions asked in the interviews were used in the validation of the concept of AARC: (1) “Over the past few years, what changes have you noticed within yourself (e.g., health, physical functioning, mindset, interpersonal relationships and personal interests)?” (2) “Have there been any incidents that made you become more aware of getting older?” (3) “Have you taken steps to counter the negative effects of aging?” (4) “Disregarding the negative effects, are you conscious of any positive sides to aging?”

As both the questionnaire and interviews were conducted in China, the English versions of the questionnaire and interview questions were translated into Chinese. Following the method of back translation (56), a Chinese PhD student in Management who is proficient in English translated the Chinese versions of questions back into English. After comparing the original and back-translated English versions of each document, we improved the translation by addressing discrepancies between them, of which only two were found. Next, a teacher of English in China, who is also a professional English–Chinese translator, checked the precision of the translations. Finally, the Chinese versions of the questionnaire and interview questions were pre-tested on Chinese master's degree students in England who had work experience. They were asked to comment on the translated questionnaire and interview questions, specifically whether any of the questions might cause misunderstandings or ambiguity. All concerns about the questionnaire and interview schedule design, such as the order of the questions, the precision of translation, and the appropriateness of the questions, were addressed before our formal fieldwork started.

### Analytic strategy

To ensure the 10-item scale of AARC was applicable to the Chinese context, we first conducted a confirmatory factor analysis. This was run using AMOS, and involved assessing five fit indices against cut-off values suggested by Kline (57): χ^2^/df (≤5), Comparative Fit Index (CFI) ≥0.90, Root Mean Square Error of Approximation (RMSEA) ≤0.08, Standardized Root Mean Square Residual (SRMR) ≤0.10, and PClose >0.05.

Second, the first stage of the validation exercise involved deductive thematic analysis of replies to the four relevant questions in the interview to find statements that connected to each of the ten items in the questionnaire. The pre-determined themes corresponded to these ten items. We then rated these statements on a scale of 1 to 5, analogous to that of the questionnaire, according to the degree to which they indicated awareness of aging, positive or negative. The two authors and a third colleague assessed these separately, and any discrepancies were then resolved. We then compared these scores to each individual's corresponding ratings in the questionnaire items, assessing the degree of similarity.

We then performed *t*-tests to evaluate the differences between (a) interviewees whose interview data was used in the validation exercise (information providers), and those whose interview did not contribute to it (non-providers), and (b) information providers and questionnaire respondents who were not interviewed (non-interviewees). A lack of significant differences between these groups would mean that views given in interviews were representative of the whole sample, strengthening our confidence that these interviews could be used to validate the questionnaire responses. Specifically, we used the independent *t*-test to assess mean differences between information providers and non-providers, and between information providers and non-interviewees.

We then used confirmatory factor analysis to establish whether the three types of proactivity were discrete. Next, we assessed the predictive power of the two dimensions of AARC, chronological age and subjective age in relation to each type of proactivity, using regression analysis. We then conducted a relative weights analysis (58) to assess the relative importance of the four age-related constructs – AARC-Gains, AARC-Losses, chronological age and subjective age – in contributing to each of the three types of proactivity. This assesses the amount of variance in the criterion variable that can be attributed to each predictor, in contrast to using the *R*^2^ which reflects the amount of variance that the predictors jointly explain. It can be used to supplement regression analysis where predictors share non-trivial amounts of variance. In our case it directly answers the research question which is the relative contribution of a newish concept compared with established ones, age and subjective age.

Finally, to test whether age is indirectly related to proactivity through the two dimensions of AARC, we performed bootstrapping to assess the significance of the mediation. We applied Hayes' PROCESS macro version 3.4 (59), using sample size of 5,000 and a 95% confidence level.

## Results

### Assessing two-factor structure of data on AARC

Our assessment of whether the data on AARC conforms to the two-factor model demonstrated that it did indeed conform. Confirmatory factor analysis revealed that a two-factor model of AARC fits the data well (χ^2^/df = 2.35, *p* < 0.001; CFI = 0.94; RMSEA = 0.06; SRMR = 0.05; *P*Close > 0.05). The two-factor structure was also compared with a single-factor model, which confirmed that a two-factor model fits the data better (one-factor model: χ^2^/df = 8.37, *p* < 0.001; CFI = 0.64; RMSEA = 0.13; SRMR = 0.11; *P*Close < 0.01). Indeed, the items constituting the second factor (relating to negative age-related change) all had low factor loadings in the one-factor model (below 0.50). Table 2 shows the results in more detail.

**Table 2:** Factor loadings of two- and one-factor models of AARC.

**Items comprising scale**	**Two-factor**		**One-factor**
	**AARC-Gains**	**AARC-Losses**	**AARC**
1) I pay more attention to my health.	0.58		0.59
2) I appreciate relationships and people much more.	0.54		0.55
3) I have more freedom to live my days the way I want.	0.25 ^a^		0.26^a^
4) I have more experience and knowledge to evaluate things and people.	0.62		0.62
5) I have a better sense of what is important to me.	0.87		0.86
6) I have less energy.		0.71	0.25
7) My mental capacity is declining.		0.72	0.10
8) I feel more dependent on the help of others.		0.04^a^	0.06^a^
9) I find it harder to motivate myself.		0.55	0.1 ^a^
10) I have to limit my activities.		0.16^a^	0.05^a^

^a^Indicates an inadequate factor loading.

However, three items in the two-factor model have factor loadings below the cut-off criterion of 0.50: one loading on the positive dimension, “I have more freedom to live my days the way I want” (λ = 0.25), and two on the negative one, “I feel more dependent on the help of others” (λ = 0.04), and “I have to limit my activities” (λ = 0.16). The latter two are also the only negative items not significantly correlated with age (0.05 and 0.07, Supplementary Table 1), which suggests that the aging process had limited influence on participants' judgements relating to these issues. The positive low-loading item may reflect family or work circumstances more than awareness of aging.

We also tested whether the three low-loading items were driven by a third, underlying factor. Confirmatory factor analysis revealed that their loadings in the related three-item factor model were not identifiable.

We removed the three low-loading items from our scales. This improves the fit (χ^2^/df = 3.15, *p* < 0.001; CFI = 0.96; RMSEA = 0.07; SRMR = 0.05; *P*Close > 0.05), confirming the reliability of the simplified scales. We also tested whether the results of any of our analyses reported below differed substantially due to the omission of these items, and they did not.

### Assessing the validity of items measuring AARC

To validate the items in the scales measuring AARC, we first identified statements in interviews corresponding to each item and rated them between one and five according to the extent to which they revealed awareness of age-related change. In Supplementary Table 2, we display examples of statements that were scored low and high for eight items. There were no statements relating to the other two items dealing with declining mental capacity and feeling more dependent on the help of others.

Our comparison between the questionnaire scores and those from the interviews revealed that 68.7% of responses were consistent (Supplementary Table 3). The majority of the discrepancies (59.4%) were between adjacent scores. If the responses are classified into three groupings by combining scores of 4 and 5, and scores of 1 and 2, leaving scores of three as the third group, the consistency percentage between questionnaire and interview responses increases to 87.3%. Significantly the item with the most divergences was the positive item with the low loading – “I have more freedom to live my days the way I want,” which questions the validity of the item, and further justifies its exclusion from our scale of AARC-Gains. The bulk of the statements relating to how much freedom respondents had over their activities referred to their limited options and constraints due to having dependents with significant care needs, children who were frequently ill or parents with health problems.

The *t*-tests to test the representativeness of the interviewers and the information providers amongst these reveals that the mean scores on each of the eight items for the information providers were largely not significantly different from those of the non-providers, and not significantly different from the non-interviewees (Supplementary Table 4). This adds to our confidence in the validity of the scale measuring AARC.

### Assessing the three-factor structure of data on proactivity

Confirmatory factor analysis of all items comprising the measures of the three types of proactivity (Table 3) revealed that a three-factor structure gives a good fit to the data (χ^2^/df = 3.31, *p* < 0.001; CFI = 0.95; RMSEA = 0.07; SRMR = 0.05; *P*Close > 0.05) and fits the data better than a one-factor structure (χ^2^/df = 9.28, *p* < 0.001; CFI = 0.82; RMSEA = 0.14; SRMR = 0.08; *P*Close < 0.01). The Cronbach's alpha for the measures of task proactivity, development proactivity and organization proactivity are 0.67, 0.82 and 0.85 respectively. The mean and standard deviation of each of the proactivity measure, and the correlations between them, are presented in Table 4.

**Table 3:** Factor loadings of one- and three-factor models of proactivity.

**Items comprising scale**	**One-factor model**	**Three-factor model**		
		**Task proactivity**	**Development proactivity**	**Organization proactivity**
1) Initiated better ways of doing your core tasks?	0.57	0.59		
2) Made changes to the way your core tasks are done?	0.72	0.76		
3) Come up with ideas to improve the way in which your core tasks are done?	0.54	0.57		
4) Developed skills which may be needed in the future?	0.67		0.81	
5) Gained experience in a variety of tasks to increase your knowledge and skills?	0.62		0.77	
6) Developed knowledge and skills in tasks critical to your future work life?	0.61		0.76	
7) Come up with ways of increasing efficiency within the organization?	0.75			0.83
8) Involved yourself in changes that are helping to improve the overall effectiveness of the organization?	0.75			0.80
9) Made suggestions to improve the overall effectiveness of the organization?	0.67			0.74
10) More devoted to passing along the knowledge you have gained than you were a few years ago?	0.66			0.63
11) More concerned with building up the next generation of employees than you were a few years ago?	0.64			0.68

**Table 4:** Mean, standard deviation (SD) and correlations for main study variables.

**Variables**	**Mean**	**SD**	**1**	**2**	**3**	**4**	**5**	**6**	**7**	**8**	**9**
1. Task proactivity	3.26	0.71	1								
2. Development proactivity	3.50	0.78	0.64***	1							
3. Organization proactivity	3.02	0.84	0.64***	0.52***	1						
4. Chronological age	40.95	8.91	0.11*	−0.06	0.23***	1					
5. Subjective age	36.86	9.44	−0.04	−0.18***	0.02	0.65***	1				
6. AARC-Gains	4.26	0.61	0.27***	0.27***	0.18**	0.14**	0.04	1			
7. AARC-Losses	3.41	0.87	−0.14**	−0.24***	−0.20***	0.16***	0.14**	0.14**	1		
8. Gender (1 = Female)	0.73	0.45	−0.17***	−0.16***	−0.13*	−0.09	−0.07	0.04	0.12*	1	
9. Organizational tenure	13.41	9.31	−0.01	−0.13**	0.09	0.65***	0.47***	0.05	0.23***	0.08	1
10. Management position	0.17	0.37	0.15**	0.16***	0.36***	0.30***	0.16***	0.09	−0.09	−0.17***	0.08

*p ≤ 0.05; **p ≤ 0.01; ***p ≤ 0.001.

### Relationships among AARC, chronological age, subjective age and proactivity

The correlations between both dimensions of AARC and chronological age are positive and significantly greater than zero (positive *r* = 0.14, *p* ≤ 0.01; negative *r* = 0.16, *p* ≤ 0.001), and although the relationship between AARC-Gains and chronological age is not strong, this suggests that older employees report greater AARC-Gains and AARC-Losses than their younger counterparts (Table 4). The correlations between the two dimensions and subjective age are insignificant and positive, respectively (positive *r* = 0.04, *p* > 0.05; negative *r* = 0.14, *p* ≤ 0.01), suggesting that the older people feel, the more they become aware of the negative aspects of aging. Chronological age and subjective age have a moderate positive correlation (*r* = 0.65, *p* ≤ 0.001), implying that the older people are, the older they feel themselves. Overall, the pattern of correlations raises no concerns about multicollinearity between our age-related concepts.

AARC-Gains is positively related to the three dimensions of proactivity, while AARC-Losses is negatively related to these three dimensions (task proactivity: positive *r* = 0.27, *p* ≤ 0.001, negative *r* = −0.14, *p* ≤ 0.01; development proactivity: positive *r* = 0.27, *p* ≤ 0.001, negative *r* = −0.24, *p* ≤ 0.001; organization proactivity: positive *r* = 0.18, *p* ≤ 0.01, negative *r* = −0.20, *p* ≤ 0.001).

Multiple regression analysis exploring the relationships amongst the four age-related constructs and three dimensions of proactivity (Supplementary Table 5) revealed that subjective age and both dimensions of AARC are significantly related to all dimensions of proactivity. AARC-Gains is positively related to all proactivity types (task proactivity: β = 0.27, *p* ≤ 0.001; development proactivity: β = 0.30, *p* ≤ 0.001; organization proactivity: β = 0.16, *p* ≤ 0.001), while AARC-Losses and subjective age are negatively related to all types (task proactivity: negative β = −0.16, *p* ≤ 0.001, subjective age β = −0.16, *p* ≤ 0.01; development proactivity: negative β = −0.23, *p* ≤ 0.001, subjective age β = −0.21, *p* ≤ 0.001; organization proactivity: negative β = −0.21, *p* ≤ 0.001, subjective age β = −0.18, *p* ≤ 0.01), suggesting that the younger a person feels, the more likely they are to be proactive. Chronological age is positively related to task proactivity and organization proactivity (task proactivity: β = 0.20, *p* ≤ 0.01; organization proactivity: β = 0.26, *p* ≤ 0.001), but not to development proactivity.

A relative weights analysis, used to allow for shared variance between the variables, showed that both AARC concepts contribute more than chronological age, subjective age, gender, organizational tenure or managerial position to predicting task and development proactivity, with the positive dimension making the strongest contribution (Table 5). In the case of organization proactivity, AARC-Losses contributes more than its positive counterpart, as does chronological age. However, managerial position predicts organization proactivity more strongly than any age-related concept. Subjective age contributes less than chronological age in predicting task and organization proactivity, but considerably more in relation to development proactivity.

**Table 5:** Relative percentage contribution of predictors of types of proactivity.

**Variables**	**Relative weights results**		
	**Task proactivity**	**Development proactivity**	**Organization proactivity**
AARC-Gains	46.17	36.71	13.25
AARC-Losses	14.26	28.50	18.36
Chronological age	9.81	2.41	16.51
Subjective age	5.58	14.06	4.25
Female	15.21	10.19	3.72
Organizational tenure	1.76	2.83	3.44
Management position	7.21	9.00	40.47
Total (%)	100	100	100

We also tested whether age is related to proactivity through its association with AARC – whether the latter mediates the effect of the former. We found the data fitted a model in which that AARC-Gains mediates the relationship between chronological age and task proactivity (β = 0.05, CI [0.01, 0.10]), development proactivity (β = 0.05, CI [0.02, 0.10]), and organization proactivity (β = 0.03, CI [0.01, 0.06]). However, the data did not fit a model in which AARC-Losses mediates these relationships (Table 6). The two types of age-related change do not therefore have counteracting effects that produce an insignificant relationship between age and proactivity. Rather, some of the relationship between age and proactivity reflects the former's association with AARC-Gains.

**Table 6:** The mediating role of both dimensions of AARC in the three chronological age–proactivity relationships.

**Mediation chain**	**Indirect effect**	**LLCI**	**ULCI**
Chronological age → AARC-Gains → Task proactivity	0.05*	0.01	0.10
Chronological age → AARC-Gains → Development proactivity	0.05*	0.02	0.10
Chronological age → AARC-Gains → Organization proactivity	0.03*	0.01	0.06
Chronological age → AARC-Losses → Task proactivity	−0.01	−0.04	0.01
Chronological age → AARC-Losses → Development proactivity	−0.02	−0.05	0.01
Chronological age → AARC-Losses → Organization proactivity	−0.02	−0.05	0.01

*Indicates a significant indirect effect.

The beta coefficients are standardized.

## Discussion

This study achieved its two objectives of validating that the concept of AARC, in both dimensions, is applicable in the Chinese context, and comparing the explanatory power of AARC, chronological age and subjective age in predicting task, development and organization proactivity. Our confirmatory factor analysis confirmed the discrete nature of its positive and negative dimensions that is consistent with other studies, and the theoretical underpinning of the AARC concept gives us some confidence that it is applicable to the Chinese context. Additionally, pilotees and respondents did not report difficulties in comprehending the questionnaire items. Our use of qualitative data to confirm their content validity is novel, and the results further suggest the items in the AARC scale can be used in the Chinese context. This also adds further support to its existing use in Western countries. Showing that the concept is valid in China suggests it may, by extension, also apply in other non-Western countries, and, given our sample, to all employed adults.

The initial factor analysis, however, revealed low factor loadings for three items in the ten-item instrument, namely: “I have more freedom to live my days the way I want,” “I feel more dependent on the help of others,” and “I have to limit my activities.” This may be due to the characteristics of the sample, which consisted of working people under 65 years old with a mean age of 41 – other studies using this 10-item scale have had samples with a higher percentage of retirees, and an average age of around 65 (6, 60, 61). We might speculate, for example, that the low loading of the item relating to increased freedom reflects that working age people must go to work to support their family and raise children, leaving little freedom, whereas retirees do not have this restriction. Similarly, the low-loading items in AARC-Losses relating to depending on others' help and having to limit activities may be due to the relatively good physical condition of our lower-aged sample, making feelings of dependency and limiting activities less likely. Past research has found that people in the UK and Canada are generally able to maintain good health before retirement (62, 63). For example, based on a meta-analysis, Pinquart (64) reported that subjective physical health decline was most likely to occur when people were over 75, and after retirement.

Analysis of the relationship between AARC and work-related proactive behavior showed that both dimensions predict all three of the proactivity types we considered, with the positive dimension positively related to them, and the negative dimension negatively related to them. Chronological age and subjective age measures also explain some variance; subjective age has a stronger association with development proactivity than the other types of proactivity, with age the more important factor in predicting organization proactivity. However, management position was by far the strongest predictor of organization proactivity. The relationship between age and all types of proactivity was found to be partly a result of its influence over AARC-Gains, but no such indirect effect was found for AARC-Losses. These results confirm that awareness of age-related change can predict outcomes beyond those related to mental health that have been at the center of its study thus far.

The significant association between AARC-Gains and AARC-Losses and types of proactivity can be explained by the dual-process model of life-course dynamics (65), which states that people shift from an assimilative mode to an accommodative mode as available resources decline, such as time, physical functioning, health, knowledge and social supports. “The assimilative mode comprises intentional efforts to modify the actual situation in accordance with personal goals, whereas the accommodative mode engages mechanisms that promote the adjustment of goals to constraints and changes in action resources” [(65), p. 117]. Displaying initiative at work is an assimilative strategy since it is a future-oriented and change-oriented behavior which aims to actively change the current situation, whereas not exhibiting or displaying proactivity to a lesser extent indicates an accommodative strategy, because employees are either not motivated to change the status quo, or do not have enough resources to reach a high level of proactivity, which requires extra time and energy. AARC-Gains and AARC-Losses reflect the level of resources people have, which influence their adoption of assimilative or accommodative strategies. People with a high level of AARC-Gains, a positive view of aging, are more likely to be adopting an assimilative strategy and therefore be proactive as they get older, which explains AARC-Gains' mediating role in the relationship between chronological age and proactivity. In contrast, AARC-Losses are associated with an accommodative strategy, explaining why people perform less proactively in this situation.

The major discrepancy between the predictors of the three work-related proactivity is that management position is highly significant for organization proactivity, but less so for the others. This may reflect the fact that managers have more authority to contribute ideas for changing the organization – indeed, a requirement to do this may be part of their job description, explicitly or implicitly. As some principals mentioned in their interviews, they were responsible for the development of the school and thus required to be proactive. Role-acceptance theory states that once people accept a role in an organization, they are also likely to accept the “specific, prescribed, often stereotypical, and impersonal expectations” resulting from the role [(66), p. 265].

Past research has found that negative psychological aging experiences are stronger than positive ones in predicting outcomes (3, 10, 15, 67). In our study this is only the case for organization proactivity. This discrepancy may be because previous research has focused on psychological aging and outcomes relating to psychomotor and physiological functioning, which are more sensitive to negative changes (3, 67), while we consider work-related proactive behaviors, which aim to improve performance, not just overcome negative feelings, and are therefore related to positive affect (68, 69) and high self-efficacy (28, 70). This difference may also possibly lie behind our finding that age may be indirectly related to proactivity through AARC-Gains, but not *via* AARC-losses.

Our research has important practical implications. First, our study implies that organizations should pay less attention to employees' chronological age, but rather take some actions to maximize positive and minimize negative experiences of aging. For example, the practice of recognizing older employees as role models in the organization can inspire them to continue to make significant contributions to their organizations. This practice may help change how they perceive themselves – they may be more aware of their positive age-related changes and feel younger, thereby promoting their proactive performance at work. Second, older people should not automatically be treated as less valuable and ripe for redundancy – instead, their potential role in organizational change should be recognized. Our findings that older employees are not less proactive than younger ones suggest that managers should disregard any negative age stereotypes and value the experience of older employees more than is typically the case, at least in China. Some research has found that Chinese firms tend to recruit younger employees due to these negative age stereotypes (71, 72). Similarly, the research adds weight to the argument that any discrimination against workers on the basis of their age needs to be avoided, and exposed and challenged where it does occur. This is a current issue in China, debated in the National People's Congress and the Chinese Political Consultative Conference in 2022 (73). It also adds to calls to increase the retirement age or the age at which pensions can be drawn in full, as has happened in some countries (74). Finally, since management position is an important predictor of organization proactivity, older employees should receive equal opportunities for promotion, not only to avoid discrimination but to foster innovation and inspire proactive behavior in people in whom it may not traditionally have been expected. Extending the autonomy given to non-managers may also help improve proactivity.

The study is limited by its cross-sectional design. With such a research design, caution is necessary in explaining the associations amongst variables, and we are unable to assess whether these indicate causality. This is also the case for mediation analysis, as there are alternative models that may also fit the data equally well (75). Third, we did not include a health variable as a control variable, partly because the ethical procedures for including this in China constrain this. Finally, since we measured proactivity by self-reports, a replication of our study could include objective measures of proactivity such as suggestions made in a particular period, to see whether AARC predicts these.

## Data availability statement

The dataset is available upon reasonable request to WZ.

## Ethics statement

The study was approved by the Research Ethics Committees of the University of Leicester (18149). The study procedures were carried out in accordance with the Declaration of Helsinki and GDPR (2018). Participants were fully informed about the aims and design of the study, so it was compliant with the GDPR (2018)'s fully-informed, opt-in consent process.

## Author contributions

WZ conducted the fieldwork and first drafted the manuscript. SW contributed to revisions. Both authors contributed to the design of the study, edited and approved the final manuscript.

## Conflict of interest

The authors declare that the research was conducted in the absence of any commercial or financial relationships that could be construed as a potential conflict of interest.

## Publisher's note

All claims expressed in this article are solely those of the authors and do not necessarily represent those of their affiliated organizations, or those of the publisher, the editors and the reviewers. Any product that may be evaluated in this article, or claim that may be made by its manufacturer, is not guaranteed or endorsed by the publisher.
