# Supplementary material for: Awareness of age-related change, chronological age, subjective age and proactivity: An empirical study in China
Source: Front Psychiatry. 2022 Sep 29;13:915673. doi: 10.3389/fpsyt.2022.915673 (PMC9558258; doi:10.3389/fpsyt.2022.915673)
Supplement: Supplementary file 1 [file Data_Sheet_1.pdf]

## Supplementary table 1: Correlations among the items measuring AARC, chronological age and subjective age

| Items | 1 | 2 | 3 | 4 | 5 | 6 | 7 | 8 | 9 | 10 | 11 |
|---|---|---|---|---|---|---|---|---|---|---|---|
| 1) I pay more attention to my health. | 1 | | | | | | | | | | |
| 2) I appreciate relationships and people much more. | 0.51*** | 1 | | | | | | | | | |
| 3) I have more freedom to live my days the way I want **(Low factor loading)**. | 0.18*** | 0.13** | 1 | | | | | | | | |
| 4) I have more experience and knowledge to. evaluate things and people. | 0.34*** | 0.34*** | 0.17*** | 1 | | | | | | | |
| 5) I have a better sense of what is important. to me. | 0.51*** | 0.47*** | 0.21*** | 0.55*** | 1 | | | | | | |
| 6) I have less energy. | 0.15** | 0.21*** | 0.05 | 0.10* | 0.19*** | 1 | | | | | |
| 7) My mental capacity is declining. | 0.07 | 0.11* | 0.06 | -0.05 | 0.06 | 0.51*** | 1 | | | | |
| 8) I feel more dependent on the help of others **(Low factor loading).** | -0.02 | -0.02 | 0.04 | -0.04 | -0.07 | 0.01 | 0.04 | 1 | | | |
| 9) I find it harder to motivate myself. | 0.10* | -0.01 | 0.14** | 0.06 | 0.05 | 0.38*** | 0.42*** | .034 | 1 | | |
| 10) I have to limit my activities **(Low factor loading).** | 0.04 | 0.02 | -0.01 | 0.01 | 0.04 | 0.11* | 0.10* | 0.11* | 0.10* | 1 | |
| 11. Chronological age | 0.04 | 0.17*** | 0.14** | 0.12* | 0.11* | 0.10* | 0.14** | 0.05 | 0.13** | 0.07 | **1** |
| 12. Subjective age | 0.00 | 0.10* | 0.05 | 0.00 | 0.01 | 0.08 | 0.16*** | 0.04 | 0.09 | 0.15** | **0.65*** ** |

Note: *p ≤ 0.05; **p ≤ 0.01; ***p ≤ 0.001 (significance levels based on two-tailed tests).

**Supplementary table 2: Examples of statements scored low and high for each of eight items used in the qualitative validation**

| Items | Low score | High score |
|---|---|---|
| **AARC-Gains** | | |
| a) I pay more attention to my health. | I think my health is OK and I haven't thought much about my diet problem and taking more exercise. Maybe the only thing I do is drinking less alcohol (Male, 33 years old, score 3). | I set up a gym at home and take exercise every evening; eat more vegetables; avoid eating food with intense flavours; eat less meat and drink less alcohol (Male, 54 years old, score 5). |
| b) I appreciate relationships and people much more. | I used to be more self-centred but now I care more for my family (Female, 32 years old, score 4). | I have very good relationships with my husband and child, and I have a family of great harmony; I think every day is wonderful now (Female, 49 years old, score 5). |
| c) I have more freedom to live my days the way I want. | I am in the sandwich generation, and I don't have much time to do what I want. It costs time caring for my parents and children (Male, 44 years old, score 2). | Since I took a back seat two years ago, I have much less work to do and have more free time. I really expect to enjoy my upcoming retirement life (Female, 52 years old, score 5). |
| d) I have more experience and knowledge to evaluate things and people. | Because I only have worked for three years, I still have a lot to learn. But I have become more mature and tolerant (Female, 29 years old, score 3). | As my life experience increases, I have my own opinions which are immune to influences from the outside world (Female, 56 years old, score 5). |

| | | |
|---|---|---|
| e) I have a better sense of what is important to me. | I feel ambivalent – sometimes I want to make more efforts to develop myself. But sometimes the idea of dawdling time away also emerges due to trivialities at work (Female, 23 years old, score 2). | I became more determined than before and nothing can change my mind (Female, 44 years old, score 5). |
| **AARC-Losses** | | |
| a) I have less energy. | Basically, I don't feel my energy is declining (Male, 45 years old, score 2). | I used to be very energetic all day long and didn't need to take a break. But since I started working two years ago, I need to stick to a schedule or I feel very tired (Female, 29 years old, score 5). |
| b) I find it harder to motivate myself. | If I don't work hard, my potential will be buried and some possibilities in the future will be gone. I want to improve myself and make something different (Male, 44 years old, score 1). | Before 30 years old, I wanted to do something in the IT industry. But as the job intensity is too high for people who are over 30, I finally chose to be a teacher and have less ideas about my future than a few years ago (Male, 33 years old, score 4). |
| c) I have to limit my activities. | I can keep up with my work and continue to do my hobbies like playing table tennis and walking (Male, 60 years old, score 2). | Because I am growing older, my increasing back pain prevents me from taking more exercise, and my declining energy really affects me doing more work for the school (Female, 50 years old, score 5). |

**Supplementary table 3: Validation results for responses which differ between questionnaire and interview**

| Questionnaire measures | Correspondence with interview themes | | |
|---|---|---|---|
| | Consistency | % | Discrepant results |
| **AARC-Gains** | | | |
| a) I pay more attention to my health. | 18/29 | 62.1 | 36 (5,4), 44a (5,4), 44b (3,4), 35 (5,4), 52 (5,4), 32b (4,1), 41 (5,4), 45 (5,4), 30 (5,4), 62 (5,1), 59 (5,4) |
| b) I appreciate relationships and people much more. | 8/8 | 100.0 | |
| c) I have more freedom to live my days the way I want. | 7/15 | 46.7 | 32b (2,1), 41 (2,1), 29b (2,1), 54 (3,4), 44a (3,4), 50b (2,4), 31a (5,4), 43b (2,4) |
| d) I have more experience and knowledge to evaluate things and people. | 16/21 | 76.2 | 50a (3,4), 36 (3,4), 44a (5,4), 44b (5,4), 31a (5,4) |
| e) I have a better sense of what is important to me. | 8/14 | 57.1 | 23 (2,3), 50c (5,4), 30 (5,4), 32a (4,5), 56a (4,5), 56b (4,5) |
| **AARC-Losses** | | | |
| a) I have less energy. | 18/24 | 75.0 | 28 (3,1), 41 (4,1), 60 (3,1), 44b (5,4), 31a (5,4), 62 (5,4) |
| b) I find it harder to motivate myself. | 10/15 | 66.7 | 44d (2,1), 35 (4,3), 23 (5,3), 43 (1,4), 32a (4,5) |
| c) I have to limit my activities. | 7/8 | 87.5 | 44c (3,2) |
| **Total** | 92/134 | 68.7 | |

Note: (1) Age is used to represent interviewees. If there is more than one interviewee with the same age, a letter is added to distinguish between. them, e.g., 30a, 30b etc.

(2) In the brackets, the first number is the questionnaire response and the second number is the subjective rating of the paired statement in the interview.

**Supplementary table 4: One-sample t-tests between information providers, non-providers and non-interviewees**

| Questionnaire measures | Mean value for information providers | Mean value for non-providers | Mean value for non-interviewees | p-value between information providers and non-providers | p-value between information providers and non-interviewees |
|---|---|---|---|---|---|
| **AARC-Gains** | | | | | |
| a) I pay more attention to my health. | 4.52 (29) | 4.50 (4) | 4.36 (388) | 0.96 | 0.25 |
| b) I appreciate relationships and people much more. | 4.75 (8) | 4.36 (25) | 4.39 (388) | 0.12 | 0.65 |
| c) I have more freedom to live my days the way I want. | 3.24 (15) | 3.67 (18) | 3.31 (388) | 0.30 | 0.71 |
| d) I have more experience and knowledge to evaluate things and people. | 4.53 (21) | 4.28 (12) | 3.93 (388) | 0.34 | 0.54 |
| e) I have a better sense of what is important to me. | 4.07 (14) | 4.00 (19) | 4.31 (388) | 0.82 | 0.57 |
| **AARC-Losses** | | | | | |
| a) I have less energy. | 3.92 (24) | 4.22 (9) | 3.91 (388) | 0.28 | 0.47 |
| b) I find it harder to motivate myself. | 2.47 (15) | 3.67 (18) | 2.54 (388) | 0.01* | 0.63 |
| c) I have to limit my activities. | 3.50 (8) | 3.84 (25) | 3.64 (388) | 0.38 | 0.51 |

Note: (1) * means there was a significant difference in mean values between the groups at the 95% significance level, with an effect size (Cohen's *d*) of -1.06.
(2) the sample sizes are presented in the brackets.

**Supplementary table 5: Multiple regression analysis of the relationships between types of proactivity, age-related constructs and control variable**

| Variables | Multiple regression | | |
|---|---|---|---|
| | Task proactivity | Development proactivity | Organization proactivity |
| AARC-Gains | 0.27*** | 0.30*** | 0.16*** |
| AARC-losses | -0.16*** | -0.23*** | -0.21*** |
| Chronological age | 0.20** | 0.04 | 0.26*** |
| Subjective age | -0.16** | -0.21*** | -0.18** |
| Female | -0.14** | -0.14** | -0.05 |
| Organizational tenure | -0.03 | -0.02 | -0.03 |
| Management position | 0.06 | 0.11* | 0.26*** |
| $R^2$ | 0.15 | 0.22 | 0.23 |
| F | 10.67*** | 16.13*** | 17.57*** |

Note: (1) *$p \leq 0.05$; **$p \leq 0.01$; ***$p \leq 0.001$.

(2) Standardized regression coefficients (beta) are reported in the table.
